# The complete chloroplast genome sequence of *Clintonia udensis* (Liliaceae)

**DOI:** 10.1080/23802359.2019.1668735

**Published:** 2019-09-23

**Authors:** Jianfang Li, Bei Xu, Qian Yang, Zhan-Lin Liu

**Affiliations:** Key Laboratory of Resource Biology and Biotechnology in Western China (Ministry of Education), College of Life Sciences, Northwest University, Xi'an, China

**Keywords:** *Clintonia udensis*, plastome, phylogeny

## Abstract

The whole chloroplast genome of *Clintonia udensis*, an import Chinese medicinal herb, was determined by Illumina sequencing data in this study. The cp genome is 153,160 bp in length, with a large single-copy region (LSC) of 83,901 bp, a small single-copy region (SSC) of 17,241 bp and a pair of inverted repeat regions (IRs) of 26,009 bp. It contains 132 genes, including 83 protein-coding genes, 36 tRNA genes, 8 rRNA genes, and 5 pseudogenes. The overall GC content was 37.3%, while the corresponding values in the LSC, SSC and IR region are 35.1, 31.0, and 42.8%, respectively. Phylogenetic analysis indicated that *Clintonia udensis* was related to Lilioideae species in Liliaceae.

Liliaceae, a large family of about 600 species, is of important economic, ecological and medicinal values. The infrafamilial relationships have not been clearly identified and produce dynamic changes along with the advantage of phylogenetic works. For example, the genus *Clintonia*, with a typical intercontinental disjunction distribution between Eastern Asia and North American, is transited from subfamily Lilioideae to subfamily Medeoloideae (Kim and Kim 2018). To explore the phylogenetic relationships of Liliales, trace the evolutionary history of the genus *Clintonia*, genome information is necessarily needed. *Clintonia udensis* Trautvetter & C. A. Meyer, mainly distributed in China, Korea, Japan, and Far East area, is a traditional Chinese medicine with the function of promoting blood circulation to remove blood stasis and treating traumatic injuries and erysipelas (Mimaki and Watanabe 2008). The cytogenetics study show that there are two ploidy levels of individuals (diploid and tetraploid) present in the natural populations of *Clintonia udensis* (Li and Chang 1996). In the present study, we determine the complete chloroplast (cp) genome of *Clintonia udensis* using Illumina sequencing data, which is expected to provide new molecular data for phylogenetic works at variable levels and population genetics studies of *Clintonia udensis* in future.

Total genomic DNA was isolated from a single individual of *Clintonia udensis* sampled from Hualong Mountains, China (N32.01°, E109.69°). The voucher (2010BAI87) was deposited at the Evolutionary Botany Laboratory (EBL), Northwest University. Read data were obtained and treated following the previous study (Peng et al. 2019). The complete chloroplast genome was annotated with *Amana edulis* (NC034707) as a reference and has been submitted to GenBank with the accession number MN136230.

The whole plastome of *Clintonia udensis* is 153,160 bp in length, with a large single-copy region (LSC) of 83,901 bp and a small single-copy region (SSC) of 17,241bp, separated by a pair of inverted repeat regions (IRs) of 26,009 bp. It contains 132 genes, including 83 protein-coding genes, 36 tRNA genes, 8 rRNA genes and 5 pseudogenes (*ycf1*, two copies of *ycf15* and *ycf68*). Sixteen genes are duplicated in the IRs, containing 5 protein-coding genes (*rpl2*, *rpl23*, *ycf2*, *ndhB*, *rps7*), 7 tRNA genes (*trnH-GUG*, *trnI-CAU*, *trnL-CAA*, *trnV-GAC*, *trnI-GAU*, *trnR-ACG*, *trnN-GUU*) and 4 rRNA genes (*rrn16*, *rrn23S*, *rrn4.5S*, *rrn5S*). Among annotated genes, fourteen (*rps16*, *atpF*, *rpoC1*, *petB*, *petD*, *rpl16*, *rpl2*, *ndhB*, *ndhA*, *trnK-UUU*, *trnG-GCC*, *trnL-UAA*, *trnl-GAU*, *trnA-UGC*) contained a single intron and three (*ycf3*, *rps12*, *clpP*) contained two introns. The overall GC content was 37.3%, while the corresponding values in the LSC, SSC, and IR region were 35.1, 31.0, and 42.8%, respectively.

In an attempt to elucidate the position and phylogenetic relationship of *Clintonia udensis* within the Liliaceae family, 17 complete chloroplast genomes from Liliales were used to construct the phylogenetic tree with the maximum-likelihood method (Peng et al. 2019) (Figure 1). The phylogenetic analysis shows that species in subfamily Lilioideae are clustered into a group and sister to subfamily Medeoloideae (including *Clintonia udensis*) with high bootstrap value, as supported by previous molecular data (Kim and Kim 2018).

**Figure 1.**
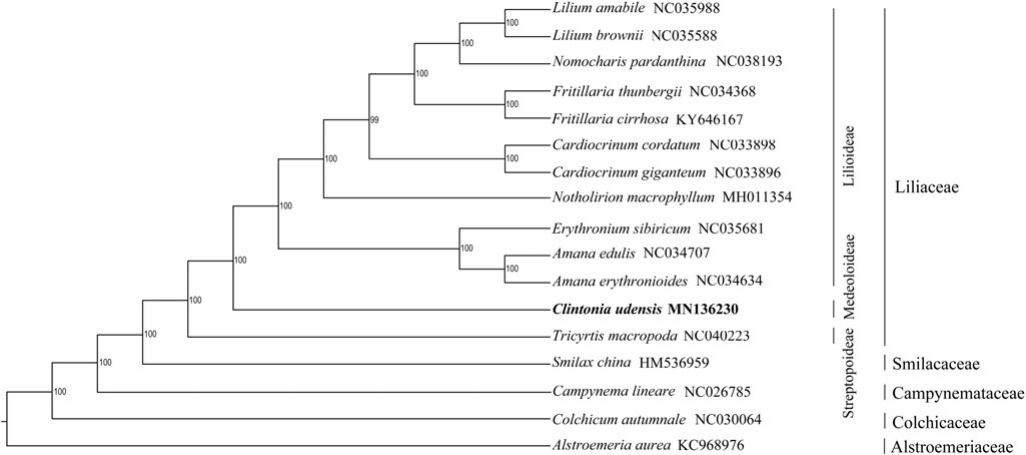
The maximum-likelihood tree based on the complete chloroplast genomes of 17 Liliales species. The bootstrap values were based on 1000 replicates.
